# Peritoneal Dialysis (PD) Patient and Nurse Preferences around Novel and Standard Automated PD Device Features

**DOI:** 10.34067/KID.0000000000000377

**Published:** 2024-02-01

**Authors:** James A. Sloand, Mark R. Marshall, Steve Barnard, Rick Pendergraft, Nick Rowland, Steve J. Lindo

**Affiliations:** 1 The George Washington University, University School of Medicine & Health Sciences, Division of Kidney Diseases & Hypertension, Washington, DC; 2 Simergent LLC, Chicago, Illinois; 3 Department of Medicine, University of Auckland, Auckland, New Zealand; 4 The Link Group, Atlanta, Georgia

**Keywords:** chronic dialysis, ESKD, patient satisfaction, patient-centered care, peritoneal dialysis, quality of life

## Abstract

**Key Points:**

- Adoption and extended time on peritoneal dialysis require patient input across a spectrum of needs, including automated PD (APD) device usability features that are less intimidating to patients and enable lifestyle advantages.
- Analysis of APD features critical to patients align with patient priorities identified in Standardized Outcomes in NephroloGy-PD: shorter setup time, mobility within the home, near silent operation, and modified APD size/orientation.

**Background:**

Despite offering greater lifestyle benefits to patients with ESKD, adoption of peritoneal dialysis (PD) remains low globally, particularly among minorities and the socioeconomically disadvantaged. While automated PD (APD) affords a high potential for reducing the burden of KRT, understanding patient preferences is critical to guiding development of new and improved APD devices to better accommodate use in their daily lives.

**Methods:**

A quantitative cross-sectional survey study was performed using adaptive conjoint analysis to quantify APD feature preferences among patients on PD, PD Registered Nurses (RNs), and non-PD patients to ascertain the relative importance of eight specific cycler attributes, including portability, noise, setup time, device size, setup directions, battery power, consumables, and PD RN control (PD RNs only), each with 2–3 descriptive feature levels.

**Results:**

Forty-two patients on PD, 24 non-PD patients, and 52 PD RNs were surveyed. Preference shares spanned nearly the entire range from 0% to 100%, indicating strong preference discrimination. For all groups, “Portability in the Home,” “Noise Level,” and “Setup Time” were the most important features. Patients on PD gave highest priority to these features compared with other study participants, plausibly as features enabling improved lifestyle. A simulated “coat rack” style cycler with extended battery power that was easy to move in the home, silent, required only 10-minute setup, and had a fully animated instruction screen was preferred by all groups >90% compared with features present in existing cyclers.

**Conclusions:**

Addressing APD cycler technical and therapy-related issues to improve usability, comfort, and convenience within the home may affect PD uptake and retention. Attention and priority must be given to patient-centric APD cycler design directed at including features that improve quality of life for the device end user.

## Visual Abstract

**Figure:**
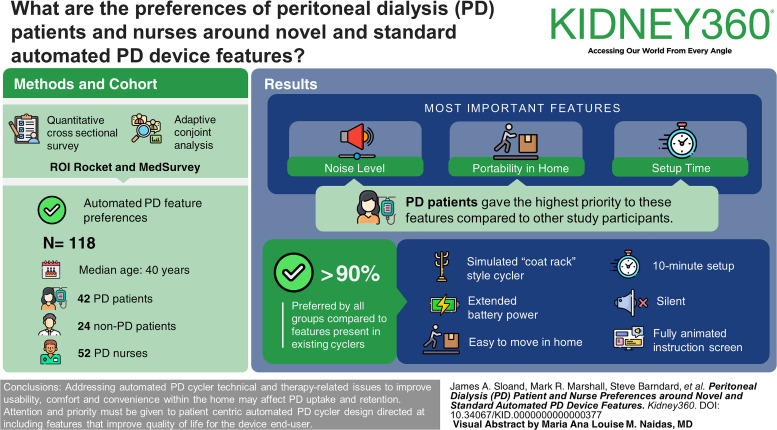

The Standardized Outcomes in NephroloGy (SONG) initiative has spearheaded a growing recognition that greater patient-centered focus is imperative in clinical trials, clinical care, and development of more patient-centric therapies.^1–3^ Patient preferences have been shown to vary significantly at times from those of health care professionals.^1–3^ As the end users of medical devices and products, patient perspectives are critical to ensure that therapy interventions address patient needs and priorities. Doing so can improve quality of daily life for patients and their care providers in the setting of chronic care needs, improve adherence to therapy, and relatedly, reduce burnout, extending time on therapy.^4^

CKD affects approximately 9% of the global population, progressing to ESKD requiring KRT in more than 2.5 million people, a number projected to double to 5.4 million by 2030.^5^ Although some patients receive kidney transplants, most patients receive either peritoneal dialysis (PD) or hemodialysis (HD).^6^ While PD, particularly automated PD (APD), offers patients with ESKD greater autonomy and lifestyle flexibility than in-center HD,^7^ PD adoption remains lower than expected, especially among minority and socioeconomically disadvantaged patients.^8,9^ Reasons for attenuated take-on of PD are multifold but include patient fear and anxiety of doing self-care as well as concerns about overall therapy complexity.^10,11^ For patients electing to do PD/APD, technique failure remains a significant problem contributed to by multiple factors.^12^ APD-related patient complaints, including suction-related drain pain and APD-related noise affecting sleep, undoubtedly also contribute to a poor patient experience and drop-out from PD.^13–15^ Solutions to these issues are imperative if the objectives of multiple Ministries of Health around the world are to be achieved.^7,16^ This includes the objectives of the Advancing American Kidney Health Initiative of 2019 to increase utilization of home dialysis and transplantation to 80% by 2025.^17^

Globally and in the United States, there is need for safe, affordable APD cycler systems that are less intimidating for patients considering home dialysis and easier for patients to learn, operate, and accommodate to use in their daily lives. In this article, we present the results of a study of patient and nurse preferences regarding the feature sets of APD cyclers. This research was conducted and sponsored by a large US health care corporation to evaluate the features of an innovative APD cycler (“Archimedes,” described as “Cycler X”) developed by a US-based dialysis manufacturer (Simergent). The device was developed by the manufacturer to address historical user complaints, injuries, and issues inherent to currently marketed APD cyclers. The aim of this study was to examine how much stakeholders value specified features of such a device compared with features of existing APD cyclers in the United States and which combination of features and aspects of the features influence them when making decisions between different devices.

## Methods

This was a quantitative cross-sectional survey study, using adaptive conjoint analysis (ACA). ACA is a form of discrete choice experiment (DCE). It is one of the standard methodologies to assess the relative importance of different product features or attributes among respondents and their preference for various combinations of them.^18^

ACA is particularly appropriate for products with potentially hundreds of combinations across many feature levels where information overload is problematic.^19^ With ACA, the respondent is not required to indicate their preferences for each and every combination because of an adaptive algorithm that limits the number of choices presented to them by using their previous choice data to eliminate irrelevant ones.^20^

The ACA approach is both well validated and accepted for its capacity to more closely predict real-world decision making than simple self-reporting or ranking of preference data among respondents. As such, it enables determination of the relative importance that respondents assign to specific features or attributes and different levels of these features or attributes.^21^ The Sawtooth software used in this manuscript (see below, Study design and data collection) has been successfully used for exploring patient preferences in cancer,^22^ degenerative joint disease,^23^ gut disease,^24^ and nephrology and dialysis medicine.^25,26^

In this study, ACA was used to evaluate several APD device features, levels of each of those features, and finally combinations of feature levels. The evaluation therefore presents decision exercises that match real life scenarios more closely by presenting various full combinations of features and asking respondents to choose one set of features over alternatives.^27^ After respondents have completed their surveys, hierarchical Bayes analysis is used to estimate preference coefficients by iteratively estimating and borrowing information from similar respondents.^28^

The recruitment, screening, and enrollment of participants as well as study procedures and data analysis were performed by an independent research organization, contracted by a large US health care company. Other than being provided results of the study and providing a list of device features to the researchers, Simergent had no role in the study.

The study was conducted, and the results are reported in accordance with the Consensus-Based Checklist for Reporting of Survey Studies (CROSS).^29^

### Sampling Frame

Participants for this study were a purposive sample from two US nationwide research panels: ROI Rocket and MedSurvey. ROI Rocket (www.roirocket.com) is a consumer panel, including approximately 2 million panel members from which the patients/caregivers were sourced. MedSurvey (www.medsurvey.com) is a health care provider panel comprised approximately 43,000 health care providers from which the nurses were sourced. As the aim of this study was to ascertain preferences of users of APD cyclers, nephrologists were not surveyed given that they rarely, if ever, directly use APD cyclers, train patients on them, or are called on to troubleshoot these device issues when patients experience usability problems.

All members opted into these panels and agreed to participate in invitation-based and incentive-based research studies. For this study, potential respondents were invited from these panels and eligibility was established by further prespecified criteria in a brief set of online screening questions before they were permitted to complete the 20-minute online survey.

Three strata of participants were enrolled, each with specific eligibility criteria as follows: PD nurses (support and manage at least six patients on PD, must regularly service PD systems, assist with patient setup and/or training on PD, or help patients troubleshoot problems with their PD), PD patients (age 18 years or older, currently on PD) and a smaller number of non-PD patients consisting of in-center hemodialysis patients (age 18 years or older, currently on in-center hemodialysis, open to switching to PD), at-home hemodialysis patients (age 18 years or older, currently on at-home hemodialysis, previously been on PD), and caregivers of PD at-home patients (age 18 years or older, communicate regularly with and is familiar with the treatment details, or is a caregiver of someone on PD).

### Study Design and Data Collection

In the online survey, each participant was presented with a series of options to choose from or rate, combining different features of a simulated APD device presented in varying order. On the basis of their previous answers, the automated survey would then generate new sets for further choice experiments until mathematical modeling requirements were met. The participant's ratings were then used to calculate the importance of each individual feature. The ACA analysis was programmed using the Lighthouse Studio 9.14.0 software package (Sawtooth Software Inc, Provo, UT).

To simplify the survey process, the rating exercise was broken down into four stages, each using 5-point anchored preference scales to ensure forced choice:

(1) Feature-level preference stage. For each simulated device feature, the participant was asked to rate the desirability of each level. These provided data on the order and magnitude of preference of the levels within each feature.
(2) Feature importance stage. For each device feature, the participant was asked to rate the importance of each feature by asking how important it was to choose between specific feature levels, allowing the model to determine their preference for each.
(3) Paired comparison trade-off stage. Participants were presented with several 2–5 feature-level combinations side-by-side and asked to choose between them.
(4) Calibration stage. Finally, participants were presented with full profile simulated APD devices with all features and levels. The system, based on responses to earlier stages, generates the profiles to represent the range of very undesirable to very desirable profiles for the participant. Participants were asked on a 0 to 100 scale how well the device profiles would fit their needs or ideals.

### Survey Characteristics

The list of features and levels included in the survey is presented in Table 1. These were chosen based on APD cyclers under development by Simergent (including Cycler X and a Point-of-Care [PoC] APD device capable of generating customized PD solutions in the home), as well as other cyclers already on the market. None of the features were mentioned as being associated with any specific APD cycler brand. Given this study's focus on user experience, the features chosen are primarily nonclinical in nature, addressing at-home ease of use, setup, and living with daily dialysis. In total, there were eight APD cycler features, each with 2–3 descriptive feature levels. Features/levels were limited to those that are already in development and did not include conceptual or speculative ones.

**Table 1:** Conjoint attributes and levels

Attributes		Levels (Features)		Short Version^a^
1	Portability	1	Easy to move around the house while doing dialysis	Easy to move
		2	Difficult to move around the house while doing dialysis	Difficult to move
		3	Unable to move around the house while doing dialysis	Unable to move
2	Noise level	1	Near silent	Near silent
		2	Occasional hums and clicks	Hums/clicks
		3	Constant white noise	White noise
3	Setup time	1	Less than 10 minutes	<10 min
		2	10–20 minutes	10–20 min
		3	Up to 30 minutes	Up to 30 min
4	Device size	1	Size of a home printer, and requires a cart or large nightstand to place solution bags on	Printer+cart
		2	Size of a coat rack, where solution bags can hang from	Coat rack
		3	Size of a home printer, and requires a second machine the size of a minifridge	Printer+minifridge
5	Directions	1	Fully animated and in color	Animated/color
		2	Not animated but in color	Motionless in color
		3	Text only	Text only
6	Battery power	1	Can run on battery power when needed	Battery
		2	Must be plugged in to run	Plugged in
7	Consumables	1	40 boxes delivered per month—requires room to store boxes between deliveries	40 boxes
		2	5 boxes delivered per month—requires plumber to connect device to home water source, daily water testing, and filter replacement every 6 months	5 boxes+water
8	Nurse control (nurses only)	1	Nurse can lock patients out of Rx changes AND have ability to bypass therapy drain phases	Lock out/bypass
		2	Nurse can lock patients out of Rx changes	Lock out only
		3	Nurse can't control either	No control

a For brevity, we will use the Short Version wording when referring to specific levels.

Six prohibitions were included to prevent illogical combinations of features and feature levels. For example, “Portability=Unable to move” was not allowed to be associated with “Device size=printer+cart or coat rack” because these sizes are intended to be more portable.

### Data Analysis

On the basis of participant ratings, part-worth utilities for each feature level were calculated using hierarchical Bayes estimation. Individual-level utility coefficients were calculated by iteratively estimating and leveraging estimates of population-level means and covariances.^30–33^ These coefficients indicate the relative importance of levels/choices within that feature *versus* other features. They represent the trade-off likelihood of choosing one level over another. As a second stage, the utilities were then loaded into a simulator that estimates preference share as it cycles through all possible product combinations of the levels: Patients on PD (396 combinations), non-PD Patients (396 combinations), and dialysis nurses (1188 combinations). Aggregate preference is calculated for each APD cycler feature level by taking the average preference share for all product combinations with that feature. Average preference share for each feature is the likelihood that a product with that feature will get chosen as preferred.

To test the preference for combinations of features in overall preference, the preference share of five full profiles were calculated for each subgroup. These resulting preference shares represent the estimated percent of the participant sample that would choose that APD cycler in each simulation. Of note, all commercially available APD cyclers in the United States, as well as the newly developed APD cycler (Cycler X) and a PoC APD concept, were mapped to create each of these full profile simulated APD devices on the basis of its bundle of specific features. Mapping of features to these cyclers was performed by the study's sponsor. Although having no ownership stake in Cycler X's manufacturer, the sponsor wanted to compare Cycler X to other commercially available cyclers to determine the former's acceptability by US patients and nurses given its gravity-based function, vertical orientation, and other distinct features.

## Results

### Participant Characteristics

One hundred eighteen participants in total were recruited and completed the online questionnaire (response rate 16%—760 recruited with 118 qualifying and completing the survey). All participants were adults and a mix of PD patients, caregivers or patients on other forms of dialysis, or PD nurses (Table 2). Forty-two percent of patients were male (27 male and 39 female patients [58%]), and the median (interquartile range) age range was 40 (24–74) years.

**Table 2:** Respondent demographics and sample sizes

Respondent Groups^a^	Sample Size	Sex (%M/F/Other)	Mean Age (SD)	Comorbidity (% Diabetes)	Comorbidity (% Hypertension)
Patients currently on PD	42	38/62/0	49.1 (12.1)	24	45
Non-PD patients	24	46/50/4	36.8 (10.8)	42	50
In-center hemodialysis patient	18	50/50/0	37.9 (11.2)	39	50
At-home hemodialysis patient	3	33/33/33	30.0 (10.0)	100	100
Caregiver of PD at-home patient	3	33/67/0	36.7 (9.9)	0	0
PD nurses	52	n/a	n/a	n/a	n/a
Total	118	41/58/2	44.6 (13.0)	30	47

M, male; F, female; PD, peritoneal dialysis; n/a, not available.

a Of the 42 PD patients, n=39 were on Automated Peritoneal Dialysis and n=3 were on Continuous Ambulatory Peritoneal Dialysis.

### Participant Preferences

#### Overall Preference Share Range

Preference share measures the probability that an attribute will be chosen over another, on all else held constant. For patients on PD, the preference range of all possible product combinations was 2.1%–98.1%, indicating strong discrimination in the level of preference for each of the features. Similarly, for non-PD patients, the range was 9.2%–94.7%. For PD nurses, the range was 3.8%–95.8%.

#### Preference Shares for Each Feature and Level

Table 3 presents preference shares for each feature level, as averaged across all product combinations that included that feature. The percentages in the far right columns of Table 3 for each level/feature of a specific attribute represent the percentage of surveyed participants indicating that a feature would be preferred by them in the context of other APD cycler attribute features. It should be noted that the total sum of percentages is not expected to sum to 100% given that choices were not mutually exclusive. Participants could indicate more than one level/feature as being preferred. Respondents could select all or none of these features as being preferred.

**Table 3:** Feature preference shares and importance ranges, by participant group

Attributes	Levels/Features and Importance Range^d^	PD Patient (n=42) (a)	Non-PD Patient (n=24) (b)	PD Nurse (n=52) (c)
Portability	Easy to move	63%	64%	63%
	Difficult to move	38%	35%	37%
	Unable to move	21%	30%^a^	26%
	Importance range	42%^e^	34%^e^	37%^e^
Noise level	Silent	80%^bc^	67%	70%
	Hums/clicks	48%	49%	49%
	White noise	36%	38%	38%
	Importance range	44%^e^	29%^e^	32%^e^
Setup time	10 minutes	61%	58%	59%
	10–20 minutes	47%	43%	46%
	Up to 30 minutes	29%	35%^a^	32%
	Importance range	32%^e^	23%^e^	27%^e^
Device size	Printer+cart	52%	49%	55%
	Coat rack	66%^bc^	57%	57%
	Printer+minifridge	33%	39%^a^	37%
	Importance range	33%^e^	18%^e^	20%^e^
Directions	Fully animated	56%	55%	57%
	Motionless	45%	45%	44%
	Text only	36%	37%	36%
	Importance range	20%^e^	18%^e^	21%^e^
Battery power	Battery powered	53%	55%	55%
	Plugged in	38%	37%	37%
	Importance range	15%^e^	18%^e^	18%^e^
Consumables	40 boxes a month	51%	47%	49%
	5 boxes a month	32%	41%^ac^	36%
	Importance range	19%^e^	6%	13%^e^
Nurse control	Lock out/bypass	n/a	n/a	55%
	Lock out only	n/a	n/a	52%
	Can't control	n/a	n/a	30%
	Importance range	n/a	n/a	25%^e^

^a,b,c^ Refers to respondent subgroups with statistically higher either preference scores or importance ranges at *P*<0.05 with the notation (*e*.*g*., ^bc^ in column “a” means that the result in column “a” is statistically greater than columns “b” and “c”). PD, peritoneal dialysis; n/a, not available.

d Importance range is the difference between the most and least preferred levels/features within an attribute. Used as an indication of the importance of an attribute.

e Indicates the importance range is significantly different than 0% at *P*<0.01 (*i*.*e*., the difference between the most and least important features within an attribute).

Within a given attribute, the importance range is defined as the difference in preference share between the most preferred and least preferred levels/features. The importance range provides a directional indication of the overall importance of that feature. For example, for patients with PD, the difference between “easy to move” at 63.5% (SD 23.5%) and “unable to move” at 21.1% (SD 15.8%) gives an importance range of 42.4%. All except one of the importance ranges shown are significantly different from 0% at *P*<0.01. Most importance ranges are also over 20%, suggesting not only a statistically significant difference but also a meaningfully stronger preference for some features over others.

In all three participant groups, “Portability in the Home” and “Noise Level” are directionally the most important features, with “Noise Level” having the largest importance range (44%) among patients with PD. Moreover, the statistical difference in importance ranges between features is also statistically significant in each group, indicating that all participants in the study have strong preference for some user-centered features in APD cyclers over others. For patients on PD, the difference between the most and least important feature (Noise Level at 43.9% versus Battery Power at 15.4%) is 28.5% (*P*<0.01). For non-PD patients (Portability at 34.1% versus Consumables at 6.0%), it is 28.1% (*P*<0.01). For PD nurses (Portability at 37.6% versus Consumables at 13.1%), it is 24.5% (*P*<0.01).

#### Full Profile Simulated APD Devices with All Features (and Levels)

As indicated in the Methods section, the calibration stage of the rating exercise generated a series of full profile simulated APD cyclers with bundled features ranging from very undesirable to very desirable on the basis of participant responses to earlier stages of the rating exercise. Table 4 presents the simulated APD cyclers with feature sets representative of all commercially available APD cyclers in the United States, as well as the newly developed APD cycler (Cycler X) and a PoC APD Concept. The table provides the preference share for these simulated devices on the basis of how well features of that simulated device profile would meet participant needs or ideals. Preference shares over 90% indicate a strong and significant preference by patients on PD and RNs for features contained in APD Cycler X. In addition, preference for APD Cycler X is significantly higher (*P*<0.001) than all four other cyclers simulated where only one of them was over 50%. These results go toward filling a high-priority unmet need as defined in the SONG-PD initiative, namely the lack of patient-centered preference data to help determine optimal therapy delivery. The results also highlight a significant gap between APD cycler features currently available on the market and those preferred by users.

**Table 4:** Simulated full profile APD cycler feature combinations and preference share

Features	APD Cycler X (a)	Conventional APD Cycler (b)	Color, Motionless UI APD Cycler (c)	Color, Animated UI APD Cycler (d)	Point of Care Cycler (e)
Portability	Easy to move	Difficult to move	Difficult to move	Difficult to move	Unable to move
Noise level	Silent	Hums/clicks	Hums/clicks	Hums/clicks	White noise
Setup time	10 minutes	10–20 minutes	10–20 minutes	Up to 30 minutes	10–20 minutes
Device size	Coat rack	Printer+cart	Printer+cart	Printer+cart	Printer+minifridge
Directions	Fully animated	Text only	Motionless	Fully animated	Fully animated
Battery power	Battery powered	Plugged in	Plugged in	Plugged in	Battery powered
Requirements	40 boxes a month	40 boxes a month	40 boxes a month	40 boxes a month	5 boxes a month
Nurse control	Lock out/bypass	Can't control	Lock out only	Lock out only	Lock out/bypass
Preference share**:** Simulated as if it is the only APD cycler available					
PD patient (n=42)	98.1% (SD 8.7%)^bcde^	28.6% (SD 31.8%)	36.0% (SD 33.7%)	29.6% (SD 31.9%)	28.5% (SD 24.1%)
Non-PD patient (n=24)	92.7% (SD 15.7%)^bcde^	20.3% (SD 25.6%)	26.6% (SD 29.2%)	30.0% (SD 31.3%)	42.5% (SD 32.7%)
PD nurse (n=52)	94.4% (SD 17.3%)^bcde^	11.1% (SD 22.9%)	42.4% (SD 33.9%)	38.3% (SD 32.3%)	51.6% (SD 31.0%)

^a,b,c,d,e^ Indicates significantly higher preference with specific cycler profiles over others at *P*<0.001 with the notation (*e*.*g*., ^bc^ in column “a” means that the result in column “a” is statistically greater than columns “b” and “c”). APD, automated PD; UI, User Interface; PD, peritoneal dialysis.

An important detail concerns the Directions attribute, which has levels pertaining to the user interface (UI). For patients on PD, a cycler with step-by-step instructions with a fully animated color screen has 20% higher preference (Table 3) than a cycler with a text-only UI (56% versus 36%, *P*<0.01). While this implies that adding a touch screen alone to a conventional APD cycler may result in approximately a 49% overall preference (28.6% from Table 4+20% extra for touch screen), it still falls far short of “APD Cycler X” at 98% preference. Notably, the bundle of contained features similar to those present in a currently available APD cycler with a fully animated color screen had only a 29.6% preference. This is most likely attributable to bundling with other less desirable features, such as longer setup time (*e*.*g*. up to 30 minutes), negatively affecting overall preference (Table 4, column d).

#### Utility and Importance Scores of Feature Levels

**Figure 1:**
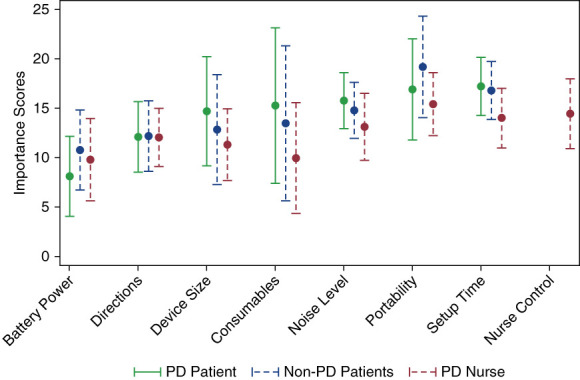
**Average importance scores for each feature, by participant group (see Appendix 2,** **http://links.lww.com/KN9/A438****).** Features displayed on the horizontal axis are ordered according to the PD patient rankings. Markers indicate the average importance, and whiskers indicate the standard deviation. PD, peritoneal dialysis.

Average utility and importance scores for each feature level are provided in Appendix 1 and 2 (http://links.lww.com/KN9/A438). These metrics supplement the directional understanding obtained from preference shares and importance ranges. The average importance scores by participant group are shown in Figure 1. Across the groups, “Noise Level,” “Portability,” and “Setup Time” ranked the highest. The only notable discrepancy was to be found in the PD nurses, for whom “Device Size,” “Consumables,” and “Noise Level” were relatively less important.

## Discussion

The findings of this study are consistent with patient-centered priorities identified in SONG-PD.^34^ One top ten priority is the preserved ability to work, study, and do home duties while receiving long-term PD. Another is for family and caregivers to be minimally affected while living with the patient and their dialysis. Our study complements these findings by adding patient-centric APD device priorities, namely shorter setup time, mobility within the home, near silent operation, and device size and orientation to enable the lifestyle priorities identified in SONG-PD.

All three participant groups had approximately similar preference for an APD cycler with greater mobility within the home, near silent operation, shorter setup time, (vertical) coat rack device orientation, an animated color GUI screen, and the availability of battery power operation when needed. However, there were notable differences in the relative importance of features between groups that suggest experiential variability. Table 3 presents that patients with PD in our study often gave the highest priority to most of the above features compared with other types of study participants. Patients on PD, whose perspective is undoubtedly influenced by “24-7” cohabitation, engagement, and at times grappling with an APD cycler, gave a directionally larger importance range between highest and lowest noise attribute level (44%) compared with non-PD patients (29%) or nurses (32%) between a “near silent” APD cycler enabled by gravity delivery versus a traditional APD cycler with “occasional hums and clicks” or “constant white noise” (Table 3). Patients on PD also gave a directionally larger importance range for reduced setup time (32%) and reduced device horizontal space occupancy (33%) as directionally more important than either PD RNs (27%/20%) or non-PD patients (23%/18%). Similarly, patients on PD were less amenable to an APD device that had the least mobility within the home (“unable to move around the house while doing dialysis” (21%)). Conversely, both PD RNs and non-PD patients, the latter group mostly composed of patients on in-center HD, found device immobility around the home (26%/30%) and larger device footprint (home printer also requiring a second minifridge-sized machine, 37%/39%) to be less objectionable than did PD patients with daily in-home PD experience (33%). Finally, as might be expected, only patients on PD (19% range) and PD RNs (13%) appreciated fewer home consumables/boxes (versus 6% for non-PD patients), although both groups had a clear trend to greater preference for enhanced device home mobility, vertical footprint, shorter setup time and, at least for PD patients, less white noise. Again, these PD patient preferences are probably owed to their “lived experience” with their APD cycler, and a consequently greater appreciation for features that might improve usability and enhance their quality of life.

There are important lessons for manufacturers from this study. The features of a taller gravity-based device described as the size of a “coat rack" scored higher preference (57%–66%) than the traditional APD cycler (49%–55%) described as the size of a “home printer and requires a cart or large nightstand to place solution bags on.” Similarly, the features of a wheeled device described as “easy to move” within the home scored higher preference (63%–64%) than one which is “difficult to move” (35%–38%) or “unable to move” (21%–30%) within the home while doing dialysis. Although APD cycler manufacturers have launched numerous tabletop APD cyclers in recent years, the horizontal footprint required for placement of PD solution bags on a cart or nightstand for use with these smaller devices has remained the standard design over the vertical orientation. Although this study did not compare cycler preference for mobility outside the home versus within the home, users clearly prefer a cycler within the home with vertical orientation and in-home mobility over horizontal devices requiring a difficult to move cart or immobile nightstand. The ability to move about the home while performing APD therapy is important when designing new APD cyclers, considering that APD therapy duration ≥9 hours in many APD populations may often exceed the duration in which patients are sleeping (Personal communication; J. Perl).^35^ This is a potential drawback of a PoC PD solution-generating APD cycler with a fixed location water filtration device and may have implications for certain home HD devices as well. Social factors, such as interaction with family members, may also play a role in this feature preference.

Another lesson is that having fewer boxes of consumables (5 boxes/month) associated with a PoC APD cycler scored lower importance (32%–41%) than having 40 boxes/month (47%–51%) among all user groups. Users were unwilling to trade off fewer boxes of consumables for lack of mobility within the home, greater noise, and increased setup time. Notably, fewer boxes of consumables associated with a PoC APD cycler concept have practical limitations, including a nonmovable water filtration device (described as similar in size to a “minifridge”), a pump and numerous valves with a speaker producing “white noise,” and the potential for increased setup time to admix PD solutions before therapy start.

The final lesson is that while user-centered design in the form of on-screen animations (*e*.*g*., GUI interface) to help patients with setup and operation are preferred over traditional text-only alternatives, this design element is not as important as those dealing with movement and convenient positioning in different parts of the house, reduced setup time, or noise. This could intuitively be thought of as the difference between usability features most beneficial during the initial learning period for incident patients versus cycler features that add more long-term value to enable enhanced patient comfort and lifestyle. Moreover, APD cycler user favorability is more powerfully influenced when such an on-screen color animated user interface is bundled with other user-preferred features facilitating movement and convenient positioning in different parts of the house, reduced setup time, or noise.

There are some limitations of this study. First, the survey was only completed by those electing to take it. Participant data about race or ethnicity of study participants were also not available. APD features were verbally described; no pictures or names of APD cyclers or their features were provided to participants taking the survey. The descriptions of new features were not formally tested to confirm that they accurately reflected the conventional cycler features or new “in-development” features. The survey also did not include costs of various APD cyclers/features, and description of battery power duration (*e*.*g*., 30 minutes versus 8+ hours) was not provided. However, the attribute of battery power had the lowest importance score of all attributes for both PD patients and nurses (Figure 1), and while an APD device that can “run on battery power when needed” was viewed as a favorable feature, it did not drive the overall favorability outcome seen. Other potential benefits or drawbacks of new cycler features were omitted (*e*.*g*., potential reduction in drain pain, reduction in peritonitis, clinical benefits of PoC solutions). Certain combinations were always paired together (*e*.*g*., mini-fridge +5 boxes/month), as the “mini-fridge” represented a potential water filtration device required for PoC solutions, or were prevented (coat rack+5 boxes/month), as this combination lacked the water filtration device required for reducing the number of boxes in the home. The results may also have varied if tested on a different patient population (*e*.*g*., from a country or region with an unreliable power grid where ability to complete a therapy with battery power might be paramount). Finally, the participants in this study were sourced from the United States only; patient perspectives in other parts of the world may be different.

There is a large patient-centered unmet need in dialysis device development in general.^36^ Many recently developed devices with strong patient focus have not been successfully brought to market.^37,38^ In addition, some ostensibly patient-centric devices designed by industry have had surprisingly poor resonance with patients after commercial release.^39^ A strong contributor to this quandary is that patient-centric design is not acknowledged by health care payers or providers and that these aspects are seen as value-added features rather than core ones.^40^ The results from this study and others highlight the importance of key device features for patients.^39,41–44^ Attention must be given to studies such as ours, given the goal of improving quality of life for the device end user. A greater understanding and adoption of patient-centric design by the dialysis community will allow more efficient product development in an industry increasingly under the pressure of narrowing margins.

## Supplementary Material
