# Supplementary material for: Peritoneal Dialysis (PD) Patient and Nurse Preferences around Novel and Standard Automated PD Device Features
Source: Kidney360. 2024 Feb 1;5(3):380–9. doi: 10.34067/KID.0000000000000377 (PMC11000714; doi:10.34067/KID.0000000000000377)
Supplement: Supplementary file 1 [file kidney360-5-380-s001.pdf]

Supplemental Data

Appendix 1

Raw utility scores for each conjoint level for each subgroup

**Attributes**	**Levels/Features**	**PD Patient (n=42)**		**Non-PD Patient (n=24)**		**PD Nurse (n=52)**	
		Avg Utilities	*Std Deviation*	Avg Utilities	*Std Deviation*	Avg Utilities	*Std Deviation*
**Portability**	Easy to move	1.70	*0.85*	1.73	*0.85*	1.79	*0.77*
	Difficult to move	-0.58	*0.55*	-0.81	*0.55*	-0.73	*0.72*
	Unable to move	-1.13	*0.80*	-0.92	*0.80*	-1.06	*0.52*
**Noise Level**	Silent	1.38	*0.62*	1.03	*0.62*	1.36	*0.57*
	Hums/Clicks	-0.10	*0.50*	0.04	*0.50*	-0.15	*0.53*
	White Noise	-1.28	*0.79*	-1.07	*0.79*	-1.21	*0.62*
**Setup Time**	10 minutes	1.49	*0.42*	1.22	*0.42*	1.34	*0.49*
	10-20 minutes	0.14	*0.24*	-0.13	*0.24*	0.05	*0.42*
	Up to 30 minutes	-1.63	*0.43*	-1.09	*0.43*	-1.40	*0.74*
**Device Size**	Printer + Cart	0.18	*1.10*	0.16	*1.10*	0.45	*0.81*
	Coat Rack	0.70	*1.12*	0.37	*1.12*	0.22	*1.14*
	Printer + Minifridge	-0.88	*0.57*	-0.53	*0.57*	-0.67	*0.80*
**Directions**	Fully Animated	1.02	*0.50*	0.87	*0.50*	1.14	*0.61*
	Motionless	-0.05	*0.35*	-0.06	*0.35*	-0.08	*0.31*
	Text Only	-0.96	*0.66*	-0.81	*0.66*	-1.06	*0.82*
**Battery Power**	Battery powered	0.75	*0.46*	0.87	*0.46*	0.92	*0.73*
	Plugged in	-0.75	*0.46*	-0.87	*0.46*	-0.92	*0.73*
**Consumables**	40 Boxes a Month	0.09	*1.61*	-0.23	*1.61*	0.08	*1.18*
	5 Boxes a Month	-0.09	*1.61*	0.23	*1.61*	-0.08	*1.18*
**Nurse Control**	Lock Out/Bypass	*n/a*	*n/a*	*n/a*	*n/a*	1.05	*0.73*
	Lock Out Only	*n/a*	*n/a*	*n/a*	*n/a*	0.62	*0.54*
	Can't Control	*n/a*	*n/a*	*n/a*	*n/a*	-1.66	*0.92*

Appendix 2

Attribute importance scores for each subgroup

**Attributes**	**PD Patient (n=42)**		**Non-PD Patient (n=24)**		**PD Nurse (n=52)**	
	Avg Importance	*Std Deviation*	Avg Importance	*Std Deviation*	Avg Importance	*Std Deviation*
**Portability**	16.91	*5.11*	19.18	*5.11*	15.42	*3.19*
**Noise Level**	15.77	*2.86*	14.78	*2.86*	13.14	*3.34*
**Setup Time**	17.21	*2.94*	16.80	*2.94*	13.99	*3.00*
**Device Size**	14.67	*5.55*	12.83	*5.55*	11.28	*3.64*
**Directions**	12.08	*3.56*	12.20	*3.56*	12.04	*2.92*
**Battery Power**	8.11	*4.04*	10.77	*4.04*	9.77	*4.13*
**Consumables**	15.25	*7.82*	13.45	*7.82*	9.95	*5.59*
**Nurse Control**	*n/a*	*n/a*	*n/a*	*n/a*	14.42	*3.54*
